# The Role of the Mitogen-Activated Protein Kinase Pathway in the Development of Laser-Induced Choroidal Neovascularization

**DOI:** 10.3390/ijms26062585

**Published:** 2025-03-13

**Authors:** Sun Young Jang, Jin Young Yang, Jin Hwan Park, Yeji Kim, Sumin An, Wook Hyun Jung, Jong-Whi Park, Jung Woo Han, Jin Ha Kim, Hyo Song Park, Jungmook Lyu, Tae Kwann Park

**Affiliations:** 1 Department of Ophthalmology, Soonchunhyang University Bucheon Hospital, Soonchunhyang University College of Medicine, Bucheon 14584, Republic of Korea; ysyat01@naver.com (S.Y.J.); 130504@schmc.ac.kr (J.H.P.); 141609@schmc.ac.kr (W.H.J.); 106236@schmc.ac.kr (J.W.H.); 114733@schmc.ac.kr (J.H.K.); 124533@schmc.ac.kr (H.S.P.); 2 Laboratory of Molecular Therapy for Retinal Degeneration, Soonchunhyang University Bucheon Hospital, Bucheon 14584, Republic of Korea; roswellgirl111@gmail.com; 3 Department of Interdisciplinary Program in Biomedical Science, Soonchunhyang Graduate School, Soonchunhyang University Bucheon Hospital, Bucheon 14584, Republic of Korea; yeiji77@naver.com (Y.K.); sue07104@gmail.com (S.A.); 4 Department of Life Sciences, Gachon University, Incheon 21936, Republic of Korea; jpakr@gachon.ac.kr; 5 Department of Medical Science, Konyang University, Daejun 32992, Republic of Korea

**Keywords:** angiogenesis, choroidal neovascularization, gliosis, inflammation, mitogen-activated protein kinase, sprouty 2

## Abstract

The role of the mitogen-activated protein kinase (MAPK) pathway in choroidal neovascularization (CNV) remains unclear. This study investigates the involvement of extracellular signal-regulated kinase (ERK), c-Jun N-terminal kinase (JNK), and p38 pathways in CNV development, as well as the therapeutic potential of sprouty 2 (SPRY2), an MAPK inhibitor, in a laser-induced mouse model. The expressions of ERK, JNK, and p38 proteins were analyzed using Western blotting and immunostaining. Immunofluorescence imaging revealed increased p-ERK and p-JNK expression in the retina, retinal pigment epithelium (RPE), and choroid up to day 7. Co-immunostaining showed p-ERK colocalized with CD31, CD11b, F4/80, cytokeratin, and GFAP in the retina, while p-JNK and p-p38 were associated with angiogenesis and inflammation throughout the retina and choroid. Compared to aflibercept, SPRY2 administration significantly inhibited CNV lesions, endothelial proliferation, fibrosis, and apoptosis, while better-preserving RPE integrity. SPRY2-treated mice showed a stronger reduction in CNV-related inflammation, epithelial–mesenchymal transition, and photoreceptor apoptosis. These results highlight the MAPK pathway’s role in CNV pathogenesis, with ERK primarily mediating Müller cell gliosis and JNK, contributing to angiogenesis and inflammation. SPRY2 effectively suppressed CNV lesions, supporting its potential as a therapeutic target for CNV treatment via MAPK pathway modulation.

## 1. Introduction

Choroidal neovascularization (CNV) refers to the abnormal growth of new blood vessels from the choroidal vasculature to the neurosensory retina through the Bruch’s membrane. This condition is observed in various retinal diseases, such as age-related macular degeneration (AMD), central serous chorioretinopathy, and pathologic myopia. CNV can lead to the leakage of fluid and blood into the retina, causing retinal damage and vision loss, particularly in cases of wet AMD, which is a leading cause of global blindness [1]. AMD is a complex and multifactorial disorder broadly categorized into two groups: dry AMD, accounting for 80–90% of cases, and wet AMD, affecting 10–15% of individuals but responsible for about 90% of AMD-related vision impairment [2,3]. While the precise pathophysiological mechanisms underlying wet AMD remain unclear, angiogenesis is recognized as a hallmark of its pathology. Consequently, anti-vascular endothelial growth factor (VEGF)-A therapy is a current treatment of choice and is widely employed for wet AMD, effectively targeting proangiogenic factors within CNV [3,4,5].

Currently, there is no definitive cure for AMD. Although the anti-angiogenic approach has shown success in inhibiting CNV development, some patients experience disease progression despite anti-VEGF treatment, suggesting the involvement of additional regulatory factors in CNV pathogenesis [6,7]. Moreover, anti-VEGF therapies have limitations that may impact normal retinal cells. VEGF plays a crucial role in the survival and maintenance of retinal pigment epithelium (RPE) integrity [8]. Research indicates that VEGF produced by the RPE is essential for balancing the choriocapillaris and the RPE complex [8], thus insufficient VEGF levels may contribute to RPE dysfunction [9]. The development of geographic atrophy, characterized by the loss of RPE in a specific area and an advanced form of dry AMD [10], may be associated with anti-VEGF therapies, prompting concerns about their long-term safety [11,12]. These limitations highlight the need to explore new treatments targeting CNV.

Mitogen-activated protein kinases (MAPKs) are a group of protein kinases that phosphorylate serine and threonine residues on their substrates [13,14]. This group includes extracellular signal-regulated kinases (ERKs), c-Jun N-terminal kinases (JNKs), and p38 kinases, which are activated by phosphorylation and play critical roles in cell proliferation, differentiation, development, transformation, apoptosis, and other cellular processes [13,14,15,16]. MAPKs have been implicated in many human diseases, including Alzheimer’s, Parkinson’s, diabetes, cancers, and AMD [15]. It is believed that MAPK signaling also plays a role in the pathogenesis of ocular diseases, such as AMD, diabetic retinopathy, retinal dystrophies, and glaucoma [13]. For instance, studies have demonstrated MAPK activation in human RPE cells following ultraviolet (UV) exposure, and oxidative stress on the RPE is thought to contribute to AMD development, with sunlight exposure believed to trigger the production of the reactive oxygen species (ROS) associated with AMD [17,18,19]. ERKs, which are part of the MAPK family, have been shown to be upregulated during photoreceptor cell death in various genetic retinal diseases, including retinitis pigmentosa and X-linked juvenile retinoschisis [20,21]. JNKs, also members of the MAPK family, play a critical role in regulating cellular processes and are activated in response to various stressors, including oxidative stress [22]. ROS are known to induce JNK activation, leading to cell death [23]. The inhibition of JNK has been demonstrated to improve hypoxia-induced retinopathy by modulating VEGF expression [24]. Furthermore, JNK inhibition reduces apoptosis and neovascularization in an AMD mouse model [25].

In the present study, we aim to investigate the role of each MAPK signaling pathway in a mouse model of laser-induced CNV. We explored how each MAPK is activated in CNV and its impact on angiogenesis, inflammation, and gliosis response. Alongside these findings, we propose sprouty 2 (SPRY2) as a potential therapeutic target for MAPK inhibition in CNV.

## 2. Results

### 2.1. Activation of ERK, JNK, and p38-MAPK Signaling in Response to CNV Induction

To explore the involvement of MAPK signaling in laser-induced CNV, we assessed the protein levels of ERK, p-ERK, JNK, p-JNK, p38 MAPK, and p-p38 MAPK in the retina, RPE, and choroid across five time points: pre-laser treatment, and 3, 5, 7, and 12 days post-laser CNV induction. In a Western blot analysis, we observed an increase in p-ERK and p-JNK proteins in response to CNV induction in the retina, RPE, and choroid. Notably, p-p38 showed significant elevation in the RPE and choroid but not in the retina.

**Figure 1:**
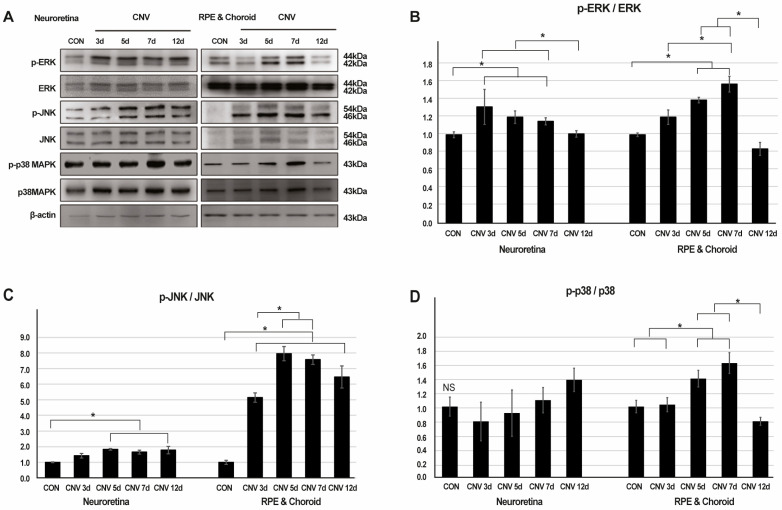
The expression levels of cytoplasmic extracellular signal-regulated kinase (ERK), phospho-ERK, c-Jun N-terminal kinase (JNK), phospho-JNK, p38 mitogen-activated protein kinase (MAPK), and phospho-p38 MAPK proteins in neuroretina, retinal pigment epithelium (RPE), and choroid at five time points: pre-laser treatment, 3, 5, 7 and 12 days after laser-induced choroidal neovascularization (CNV). (**A**) Representative Western blot images. (**B**–**D**) Mean band intensities of western blot analysis calculated using ImageJ for phospho-ERK/ERK, phospho-JNK/JNK, and phospho-p38 MAPK/p38 MAPK protein ratios (* *p* < 0.05, NS: not significant).

Specifically, the p-ERK/ERK ratio progressively increased until 7 days post-laser CNV induction in the RPE and choroid, with statistically significant differences observed (1.00 ± 0.02, 1.20 ± 0.08, 1.40 ± 0.03, 1.58 ± 0.09, and 0.84 ± 0.08, respectively, *p* = 0.015). Conversely, these differences were not significant in the retina (1.00 ± 0.03, 1.32 ± 0.20, 1.20 ± 0.07, 1.15 ± 0.04, and 1.01 ± 0.04, respectively, *p* = 0.23) (Figure 1A,B, Figures S1 and S2). The p-JNK/JNK ratio showed a similar trend, increasing until 5 days post-laser CNV induction in the RPE and choroid, with significant differences noted (1.00 ± 0.13, 5.14 ± 0.30, 7.96 ± 0.46, 7.57 ± 0.31, and 6.46 ± 0.71, respectively, *p* < 0.001). In contrast, the retina exhibited less-pronounced changes (1.00 ± 0.03, 1.42 ± 0.15, 1.83 ± 0.05, 1.66 ± 0.12, and 1.78 ± 0.24, respectively, *p* = 0.14) (Figure 1A,C, Figures S1 and S2). Furthermore, the p-p38/p38 ratio increased until 7 days post-laser CNV induction in the RPE and choroid, with significant differences observed (1.00 ± 0.09, 1.03 ± 0.10, 1.40 ± 0.12, 1.62 ± 0.15, and 0.79 ± 0.06, respectively, *p* = 0.007). Conversely, no significant changes in p-p38 were observed in the retina (1.00 ± 0.13, 0.79 ± 0.27, 0.91 ± 0.33, 1.09 ± 0.18, and 1.38 ± 0.17, respectively, *p* = 0.20) (Figure 1A,D, Figures S1 and S2).

**Figure 2:**
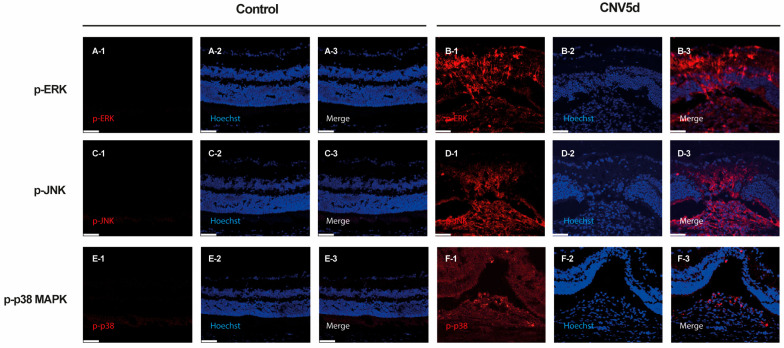
Immunocytochemical staining of (**A**,**B**) phosphorylated extracellular signal-regulated kinase (ERK), (**C**,**D**) c-Jun N-terminal kinase (JNK), and (**E**,**F**) p38 mitogen-activated protein kinase (MAPK) in cells from cryosections of the mouse retina with laser-induced choroidal neovascularization (CNV) lesions at 5 days. Scale bar = 50 µm.

To characterize the distribution pattern of activated MAPK signaling, immunohistochemistry of p-ERK, p-JNK, and p-p38 was conducted on cryosections of retina and choroid from mice sacrificed 5 days post-laser injury and controls. Immunostaining revealed increased expression of p-ERK, p-JNK, and p-p38 following laser CNV induction compared to controls. Specifically, p-ERK showed strong expression throughout the retina and moderate expression in the choroid. P-JNK exhibited widespread strong expression in both the retina and choroid, while p-p38 was primarily localized to the choroid with moderate expression (Figure 2).

### 2.2. ERK, JNK, and p38-MAPK Signalling Pathway Differentially Induce Angiogenesis, Fibrosis, Inflammation, and Gliosis After Laser-Induced CNV

All cell types involved in CNV development, namely endothelial cells, fibroblasts, inflammatory cells, and glial cells, were evaluated using immunostaining with markers CD31, α-SMA, CD11b, F4/80, cytokeratin, and GFAP.

**Figure 3:**
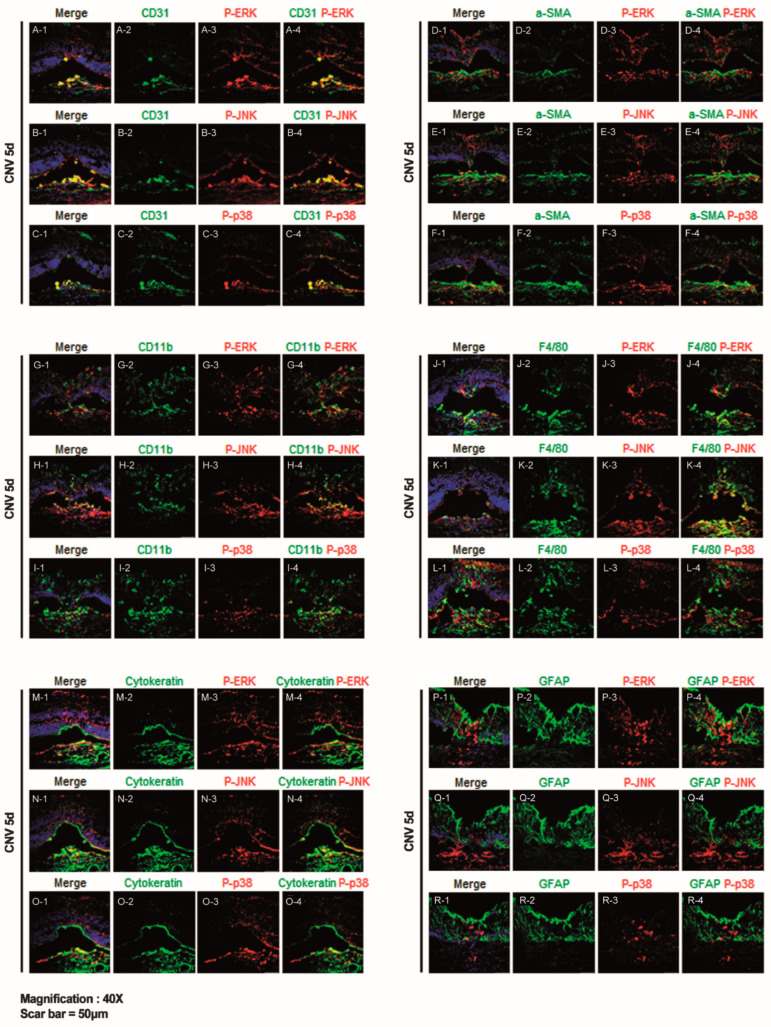
Representative confocal images of cryosections obtained on day 5 after laser photocoagulation co-stained for markers related to angiogenesis, inflammation, and gliosis, including phospho-extracellular signal-regulated kinase (ERK), phospho-c-Jun N-terminal kinase (JNK), and phospho-p38. Immunofluorescence staining for CD31 with (**A**) phospho-ERK, (**B**) phospho-JNK, and (**C**) phospho-p38. Scale bar = 50 µm. Immunofluorescence staining for α-SMA with (**D**) phospho-ERK, (**E**) phospho-JNK, and (**F**) phospho-p38. Scale bar = 50 µm. Immunofluorescence staining for CD11b with (**G**) phospho-ERK, (**H**) phospho-JNK, and (**I**) phospho-p38. Scale bar = 50 µm. Immunofluorescence staining for F4/80 with (**J**) phospho-ERK, (**K**) phospho-JNK, and (**L**) phospho-p38. Scale bar = 50 µm. Immunofluorescence staining for cytokeratin with (**M**) phospho-ERK, (**N**) phospho-JNK, and (**O**) phospho-p38. Scale bar = 50 µm. Immunofluorescence staining for glial fibrillary acidic protein (GFAP) with (**P**) phospho-ERK. (**Q**) phospho-JNK, and (**R**) phospho-p38. Scale bar = 50 µm.

In cryosection of the retina/RPE/choroid/sclera complex following laser injury (Figure 3), we observed CD31-positive endothelial cells prominently co-localized with p-ERK, p-JNK, and p-p38-expressing cells at 5 days post-laser CNV induction. CD31-positive endothelial cells were primarily detected at the inside of CNV lesions, with sparse distribution in the retina. Among the cellular components of CNV, CD31-positive cells co-localized with cells expressing p-ERK, p-JNK, and p-p38. (Figure 3A–C), suggesting that neovascularization primarily occurred within the choroid via the ERK, JNK, and p38 signaling pathway.

α-SMA-positive cells, indicative of fibroblasts, also co-localized with p-ERK-, p-JNK-, and p-p38-expressing cells at 5 days post-laser CNV induction (Figure 3D–F). Similar to CD31-positive endothelial cells, α-SMA-positive fibroblasts were primarily detected at the inside of the CNV lesion, with sparse distribution in the retina (Figure 3D–F).

Furthermore, CD11b-positive cells, representing inflammatory cells, co-localized with p-ERK and p-JNK expressing cells at 5 days post-laser CNV induction (Figure 3G–I). F4/80-positive cells, indicative of murine macrophages, also co-localized with p-ERK- and p-JNK-expressing cells. Both CD11b and F4/80-positive cells were distributed throughout the retina and particularly within the CNV lesion area (Figure 3J–L).

Cytokeratin-positive cells, indicative of epithelial cells, co-localized with p-ERK-, p-JNK-, and p-p38-expressing cells at 5 days post-laser CNV induction (Figure 3M–O). Cells expressing p-ERK, p-JNK, and p-p38 were distributed throughout the retina and choroid, co-localizing with cytokeratin-positive cells (Figure 3M–O).

Additionally, we assessed the immunoreactivity of GFAP-positive cells, a marker of retinal gliosis, with the MAPK pathway in cryosections from CNV mice sacrificed on day 5, using p-ERK, p-JNK, and p-p38 immunostaining. Confocal imaging revealed increased co-immunoreactivity of GFAP-positive cells, with p-ERK in the inner and outer nuclear layers (Figure 3P–R), suggesting that the retinal gliosis of Müller cells in these regions was mediated via the specifically ERK signaling pathway.

### 2.3. SPRY2 Reduce CNV Induction by Inhibiting Endothelial Cell Proliferation, Epithelial–Mesenchymal Transition (EMT), and Apoptosis

**Figure 4:**
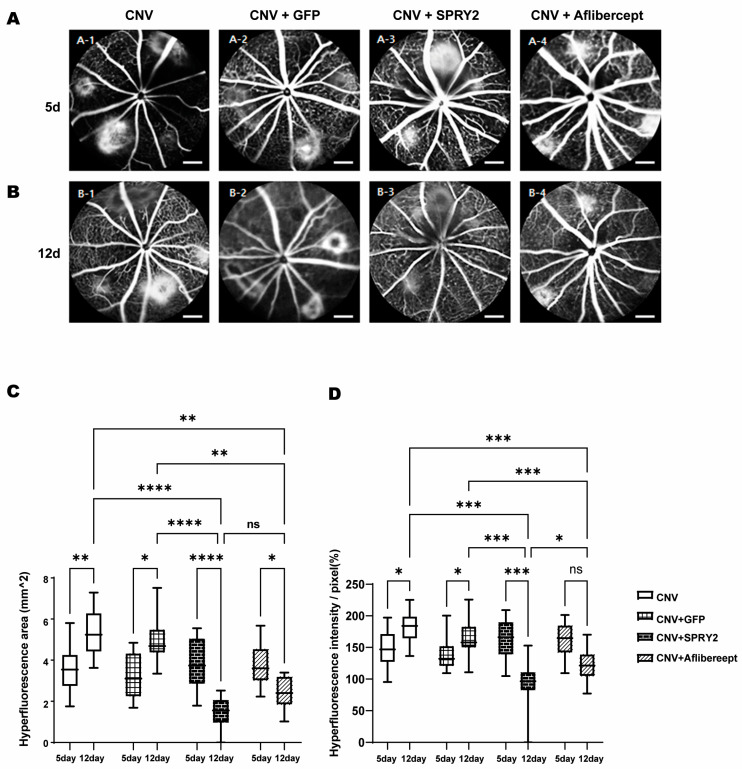
Effects of sprouty 2 (SPRY2) and aflibercept on choroidal neovascularization (CNV) formation in fundus fluorescein angiography (FFA) images. (**A**,**B**) Representative fluorescein angiograms of mice treated with CNV + green fluorescent protein (GFP), CNV + SPRY2, and CNV + aflibercept mice at 5 days after laser photocoagulation. (**C**,**D**) Quantitative analysis of FFA images for hyper-fluorescence area and intensity value. Data are presented as the mean ± standard deviation. (* *p* < 0.05, ** *p* < 0.001, *** *p* < 0.0005, **** *p* < 0.0001, and ns: not significant). Scale bar = 200 µm.

FFA images were taken at 5 days (Figure 4A) and 12 days (Figure 4B) after laser CNV induction to evaluate the effect of SPRY2 on the visible CNV area. Well-demarcated hyper-fluorescent signals were observed around the optic disc at 5- and 12-days post-laser CNV induction (Figure 4A,B). However, the hyper-fluorescent signals decreased at 12 days in SPRY2 and aflibercept-treated mice (Figure 4B). In this experiment, an intravitreal injection of AAV2-EF1a-EGFP was used as a negative control (CNV + GFP), while an intravitreal injection of aflibercept served as the positive control (CNV + Aflibercept) (Figure 4A,B). An analysis of FFA images confirmed a statistically significant decrease in the hyper-fluorescent area and intensity at day 12 compared to day 5 in SPRY2 and aflibercept-treated mice (Figure 4C,D). Compared to the day 12 control group, the SPRY2-treated mice showed a significant reduction in hyper-fluorescent area and intensity, whereas the aflibercept-treated mice showed a reduction that was not statistically significant (Figure 4C,D).

**Figure 5:**
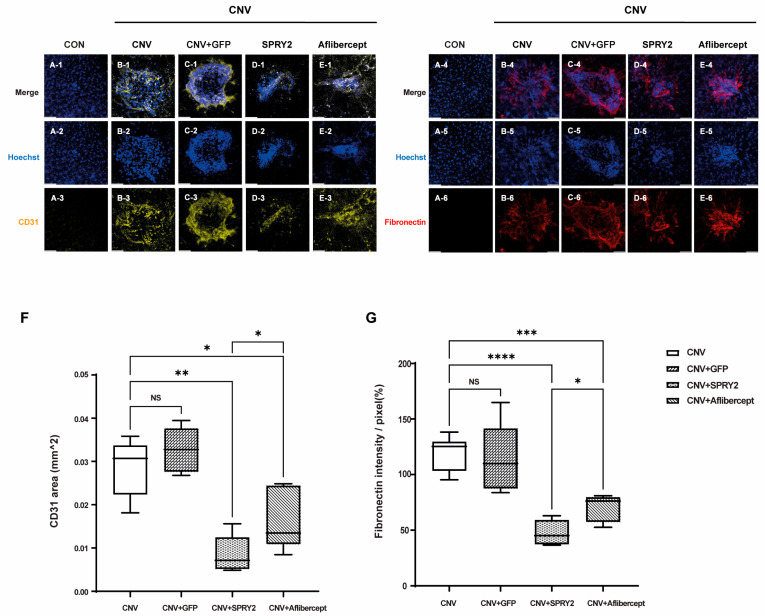
Immunocytochemical staining of CD31 + cells and fibronectin from whole-mount preparations in control, choroidal neovascularization (CNV) 5d, CNV + green fluorescent protein (GFP), CNV + sprouty 2 (SPRY2), and CNV + aflibercept mice at 5 days after laser photocoagulation. (**A**–**E**) In SPRY2 and aflibercept-treated mice, CD31 (+) area was significantly decreased compared to CNV 5d and CNV + GFP groups. (**F**,**G**) In SPRY2 and aflibercept-treated mice, fibronectin (+) area was significantly decreased compared to CNV 5d and CNV + GFP groups. (* *p* < 0.05, ** *p* < 0.001, *** *p* < 0.0005, **** *p* < 0.0001, and NS: not significant). Scale bar = 50 µm.

**Figure 6:**
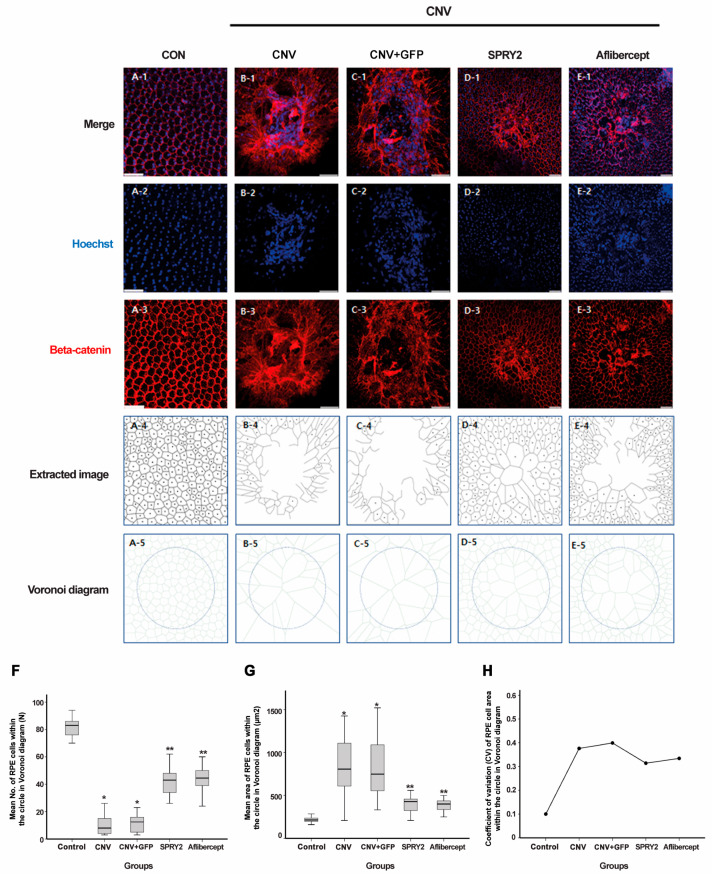
Effects of sprouty 2 (SPRY2) and aflibercept on cell-to-cell junction of retinal pigment epithelial (RPE) cells. (**A**–**E**) Morphology of RPE cells using β-catenin immunohistochemistry staining from whole-mount preparation in control, choroidal neovascularization (CNV) 5d, CNV + green fluorescent protein (GFP), CNV + sprouty 2 (SPRY2), and CNV + aflibercept mice at 5 days after laser photocoagulation. (**F**–**H**) Mean number, mean area, and coefficient of variance (CV) of RPE cell areas calculated with Voronoi diagram within a circle of 200 µm. Scale bar = 50 µm, (* *p* < 0.05, ** *p* < 0.001).

Furthermore, we evaluated the effect of SPRY2 on endothelial cell proliferation and fibrotic changes in RPE cells after laser CNV induction. Immunocytochemical staining of CD31-positive cells and fibronectin from whole-mount preparation in control, CNV 5d, CNV +GFP, CNV + SPRY2, and CNV + Aflibercept mice at 5 days after LP were performed. CD31 expression was intense in the CNV 5d and CNV +GFP group (Figure 5(B-3),(C-3)). Confocal images of the whole mounts demonstrated decreased expressions of CD31 inside the CNV lesion at the level of the RPE layer in CNV + SPRY2, CNV + Aflibercept mice (Figure 5(D-3),(E-3)). These differences were statistically significant with respect to the CD31-positive area (μm^2^) (Figure 5F), with a greater reduction in the CD31-positive area observed in the SRY2-treated group compared to the aflibercept-treated group. Furthermore, we observed fibronectin, a fibrotic marker. Fibronectin was not expressed in the control group (Figure 5(A-6)), whereas it was expressed at the center of the laser spots in other groups (Figure 5(B-6)). In the CNV and CNV + GFP groups, fibronectin expression was higher. In SPRY2 and aflibercept-treated mice, fibronectin expression decreased compared to the center of the laser-induced lesions (Figure 5(D-6),(E-6),G). Importantly, SPRY2-treated mice exhibited a greater reduction in fibronectin expression compared to aflibercept-treated mice (Figure 5G).

To evaluate the effect of SPRY2 on structural changes in RPE cells following laser CNV induction, we assessed the cell-to-cell junctions in RPE cells using β-catenin IHC staining 5 days post-laser application. Regular hexagonal β-catenin structures were absent at the center of the laser-induced lesions (Figure 6A–C). Compared to the CNV and CNV + GFP groups (Figure 6B,C), the center of the laser CNV lesion in aflibercept-treated mice showed partial coverage by RPE cells (Figure 6E). In SPRY2-treated mice, the center of the laser CNV lesion was covered with RPE cells, with the cell-to-cell connections almost fully restored (Figure 6D). RPE cells were better preserved in SPRY2-treated mice compared to those treated with aflibercept (Figure 6D,E). To assess the phenotype changes in the restored RPE cells, we analyzed the mean number of RPE cells within a circle, individual RPE cell areas using Voronoi diagrams, and the variation in RPE cell size (coefficient of variance [CV]) within a circle that was 200 µm of laser-treated area (before LP and 5 days after LP). At 5 days post-LP, SPRY2- and aflibercept-treated mice demonstrated a significant increase in the mean number of RPE cells within the circle, a significant decrease in the mean area of individual RPE cells, as shown in Voronoi diagrams, and a significant reduction in the CV of the RPE cell areas (Figure 6F–H).

**Figure 7:**
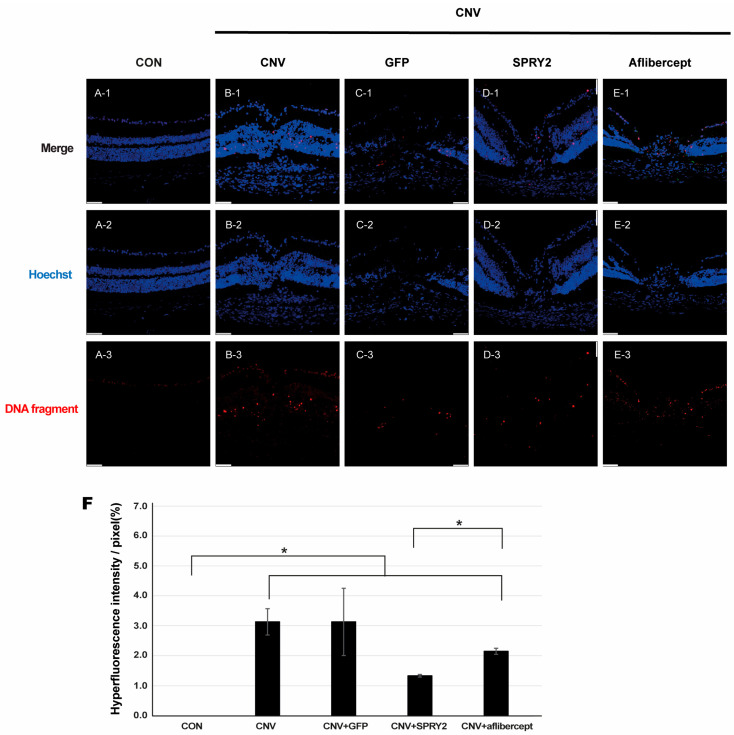
Terminal deoxynucleotidyl transferase dUTP nick end labeling (TUNEL) assay. Immunocytochemical staining of DNA fragments in cells from cryosections of (**A**) control, (**B**) choroidal neovascularization (CNV) 5d, (**C**) CNV + green fluorescent protein (GFP), (**D**) CNV + sprouty 2, and (**E**) CNV + aflibercept mice at 5 days after laser photocoagulation. Scale bar = 50 µm. (**F**) Quantification of TUNEL-positive cells, demonstrating the anti-apoptotic effect of sprouty 2 (* *p* < 0.05).

To evaluate the effect of SPRY2 on RPE cell apoptosis after laser application, we performed a TUNEL assay using cryosection of neuroretina preparation in the control, CNV, CNV +GFP, SPRY2, Aflibercept mice at 5 days after LP (Figure 7). DNA fragments were not expressed in the control group (Figure 7(A-3)), whereas they were expressed at the center of the laser spot in the other groups (Figure 7B–E). Notably, SPRY2-treated mice showed a greater reduction in DNA fragment expression, especially in the photoreceptor layer compared to aflibercept-treated mice (Figure 7(D-3),(E-3),F).

## 3. Discussion

The exact role of the MAPK pathway in the development of CNV remains unclear. In this study, we aimed to investigate the involvement of the ERK, JNK, and p38 pathways in the development of laser-induced CNV. Our results indicated that the expression of p-ERK and p-JNK proteins increased in response to laser CNV induction up to day 7 in both the entire retina and the RPE/choroid complex. Additionally, we observed a significant increase in p-p38 expression in the RPE and choroid, but not in the retina. Although the involvement of the MAPK pathway in AMD has been continuously studied, the precise mechanisms linking MAPK and CNV remain complex. Consistent with our findings, phosphorylated ERK1/2, JNK, and p38 were all found to be upregulated in a very low-density lipoprotein receptor knockout mouse (Vldlr−/−), which is a different mouse model of wet AMD [26]. Similarly, the expression of ERK1/2, JNK, and p38 was shown to be upregulated by exposure to light in a light-induced retinal damage model in adult rats [27]. Another study showed that ERK1/2, JNK, and p-38 were phosphorylated and activated mainly in the retinal outer nuclear layer (ONL) in a rat model of light-induced retinal degeneration [28]. In the present study, we used immunofluorescence to examine the expression and location of MAPKs in laser-induced CNV. We found that activated ERK, JNK, and p38 were expressed throughout the chorioretinal region. P-ERK, p-JNK, and p-p38 were activated during CNV development, both within and outside the CNV lesions, although their expression patterns were different (Figure 2). This suggests that they might have different roles depending on their activation locations.

Immunofluorescence images in the cryosections of the retina/RPE/choroid/sclera complex 5 days after laser-induced CNV revealed distinct patterns of p-ERK, p-JNK, and p38 colocalization with all cell types involved in CNV developments, namely endothelial cells, fibroblasts, inflammatory cells, and glial cells (Figure 3). P-ERK-positive cells showed co-localization with CD31, α-SAM, CD11b, F4/80, cytokeratin, and GFAP-expressed cells primarily in the retinal layer, while p-JNK and p-38 positive cells were co-localized with these markers except GFAP throughout the retina and choroid. GFAP-positive retinal gliosis markers showed greater co-immunoreactivity with p-ERK in the inner and ONLs, suggesting gliosis, especially via the ERK pathway. These results support the hypothesis that ERK is primarily involved in Müller cell gliosis primarily in the retina, whereas JNK and p38 significantly influence pathological angiogenesis and inflammation in the CNV lesion area. Consistent with our findings, Tezel et al. [29] investigated the association of MAKPs with gliosis in glaucoma, observing that retinal astrocytes and Müller cells exhibited hypertrophic morphology and increased GFAP immunostaining in the glaucomatous retina. They noted an elevated expression of p-ERK, specifically in the glial cells, while p-JNK and p-p38 immunostaining was predominantly found in nonglial cells [29]. Similarly, Gao et al. [30] reported that GFAP expression was primarily detected in the Müller cells associated with p-ERK rather than p-JNK or p-p38 in rat retinas. In normal retinas, the intravitreal injection of BaCl2 significantly increased GFAP expression in the Müller cells, which was eliminated by co-injecting the MAPK inhibitor U0126. They found significant increases in p-ERK1/2 and its upstream regulator, p-MEK, whereas the levels of p-JNK and p-p38 remained unchanged [30]. Additionally, Zeng’s research [31] demonstrated that inhibiting p-ERK reduced GFAP expression in Müller cells and contributed to the protection of the photoreceptor. Therefore, the upregulation of GFAP in various pathological disorders indicates gliosis, and p-ERK is thought to be involved in Müller cell gliosis.

JNK has been demonstrated to activate numerous substrates, influencing processes such as apoptosis, proliferation, tumorigenesis, angiogenesis, and inflammation. This activation occurs in response to various stimuli, including cytokines, pathogens, growth factors, oxidative stress, ultraviolet radiation, toxins, and drugs [32,33]. Indeed, studies using a JNK inhibitor to treat AMD have already been conducted. In a murine model of wet AMD, the mice lacking JNK1 showed reduced inflammation, decreased CNV, lower choroidal VEGF levels, and impaired choroidal macrophage recruitment [25]. Similarly, in a murine model of retinopathy of prematurity, JNK1-deficient mice exhibited reduced pathologic angiogenesis and lower retinal VEGF production [24]. This suggests that JNK plays a significant role in pathologic inflammation and angiogenesis.

Previous studies have demonstrated the involvement of MAPK in AMD via multiple contexts. Because excessive oxidative stress, apoptosis, angiogenesis, and inflammation are important pathological mechanisms in AMD, studies elucidating the involvement of the MAPK pathway in AMD have focused on its role in these processes. For instance, some studies have investigated the protective effects on RPE cells in an in vitro model of UV-induced damage by inhibiting UV-induced ERK, JNK, and p38 activation [19,34]. Another study reported that the ERK pathway, but not the JNK pathway, was involved in oxidative stress-induced apoptosis in RPE cells [35]. Because RPE cells play important and multiple roles in the retina, the majority of in vitro studies of AMD use RPE cells. Furthermore, findings from three extensive genotyping projects involving 1177 individuals with advanced AMD and 1024 AMD-free elderly individuals indicated that JNK signaling contributed to the risk of advanced AMD [36].

SPRY proteins are inhibitors of receptor tyrosine kinase (RTK) signaling, with SPRY 1–4 playing crucial roles in embryonic development [37]. Among the four mammalian SPRY homologs (SPRY1–4), overexpressed SPRY1 and SPRY2 inhibit VEGF–induced proliferation and differentiation by repressing pathways that activate MAPK [37]. Specifically, SPRY2 exerts its inhibitory effect on the Ras/MAPK pathway by targeting the activation of Raf [38]. In their study, the authors investigated the impact of various mammalian SPRY isoforms on the Ras/MAPK pathway in cells with constitutively active fibroblast growth factor receptor 1. They found that human SPRY2 (hSPRY2) exhibited a significantly stronger inhibition of the Ras/MAPK pathway compared to murine SPRY1 (mSPRY1) or mSPRY4. Notably, hSPRY2 did not affect the JNK or p38 pathway, nor did it inhibit FRS2 phosphorylation, Akt activation, or Ras activation [38]. SPRY2 inhibits the RAS/MAPK/ERK pathway and is a potential study target for colorectal cancer [39] and IgG nephropathy [40]. In the retina, SPRY2 appears to play a significant role in eye development, regulating the positioning of retinal progenitors by suppressing the Ras/Raf/MAPK pathway [41].

In the present study, FFA images, cell-to-cell junction of RPE cells, TUNEL assays, CD31, and fibronectin IHC were performed after applying SPRY2 to evaluate the effect of MAPK pathway inhibition on CNV development. Our results showed significant reductions in CNV size and leakage. The cell-to-cell junctions of RPE cells were better preserved in the SPRY2 group compared to the aflibercept and CNV groups. Apoptotic cells (DNA fragments) were scarcely observed in the SPRY2 group compared to the aflibercept and control group, suggesting a potential protective effect of SPRY2 against photoreceptor cell death. Additionally, we evaluated the effect of SPRY2 on fibrotic changes in RPE cells; fibronectin expression was decreased in SPRY2-treated mice. Notably, in terms of protection against laser-induced CNV, SPRY2-treated mice exhibited superior outcomes compared to those treated with aflibercept, which is the current treatment of choice for AMD. While the therapeutic effect of aflibercept on CNV reduction is likely attributable to its anti-VEGF properties, SPRY2 appears to act through inhibition of the MAPK pathway, specifically via ERK, JNK, and p38. This mechanism may not only inhibit angiogenesis but also address fibrosis and potentially reduce oxidative damage to RPE cells caused by ROS. Additionally, EMT inhibition and the potential prevention of photoreceptor cell death may contribute to SPRY2’s effect. These findings suggest that SPRY2 could address a broader range of AMD-related pathologies that cannot be resolved through anti-VEGF treatments alone. Consequently, SPRY2 might also inhibit geographic atrophy, positioning it as a potential alternative therapeutic option to aflibercept.

In conclusion, the results demonstrate the significant involvement of the MAPK pathway, particularly the ERK and JNK pathways, in the development of CNV. Immunofluorescence imaging revealed that p-ERK-positive cells were closely associated with markers such as CD31, CD11b, F4/80, and GFAP mainly in the retinal layer, while p-JNK-positive cells showed colocalization with CD31, CD11b, and F4/80 throughout the retina and choroid. ERK is primarily involved in Müller cell gliosis, primarily in the retina, whereas JNK significantly influences pathological angiogenesis and inflammation in both the retina and CNV lesion area. The administration of SPRY2 effectively inhibited CNV lesions, highlighting its therapeutic potential. These findings suggest that targeting the MAPK pathway could be a promising strategy for the treatment of CNV.

## 4. Materials and Methods

### 4.1. Animal Care and Use

Animal care was conducted in accordance with the guidelines of the Guide for the Care and Use of Laboratory Animals, the Association for Research in Vision and Ophthalmology Statement for the Use of Animals in Ophthalmic and Vision Research. The study protocol was approved by the Institutional Animal Care and Use Committee of Soonchunhyang University Bucheon Hospital (SCHBCIBC 2023-003). Healthy 8-week-old male C57/BL6 mice (OrientBio, Seongnam, Republic of Korea) were used in this experiment. The room temperature was maintained between 23–26 °C, and the humidity was kept at 50%. Mice were housed in breeding cages under a 12-h light/dark cycle with ad libitum access to food and water.

### 4.2. Induction of CNV in Mice

To induce CNV in the mouse retina, the following procedures were performed. Mice were anesthetized by intraperitoneal (IP) injection of a mixture containing 40 mg/kg zolazepam/tiletamine (Zoletil 50; Virbac, Carros, France) and 5 mg/kg xylazine (Rompun; Bayer Healthcare, Leverkusen, Germany). The pupil was dilated by instilling a mixture of 0.5% tropicamide and 0.5% phenylephrine (Tropherine; Hanmi Pharm, Seoul, Republic of Korea).

Laser photocoagulation (LP) was performed using a PASCAL diode ophthalmic laser system (neodymium-doped yttrium–aluminum–garnet [Nd:YAG], 532 nm; Topcon Medical Laser Systems, Livermore, CA, USA) with the parameters set as follows: 200 μm spot size, 20 ms duration, 150 mW laser power. Five laser spots were applied per eye at the 2, 4, 7, 10, and 12 o’clock positions around the optic disc, focusing on the RPE. Successful disruption of Bruch’s membrane was confirmed by the presence of a bubble at the laser application site.

### 4.3. Experimental Design

Thirty mice were assigned to five groups based on the day of sacrifice after CNV induction: (1) normal group; (2) CNV 3d; (3) CNV 5d; (4) CNV 7d; and (5) CNV 12d [42]. Additionally, forty mice were divided into four groups: (1) CNV12d, (2) CNV 5d + AAV2-EF1a-EGFP, intravitreal injection 7d, (3) CNV 5d + AAV2-EF1a-LoxP-SPRY2-2A-EGFP, intravitreal injection 7d, and (4) CNV 5d + Afilbercept 7d.

Five days after LP, CNV formation was confirmed in all mice through fundus fluorescein angiography (FFA) imaging. Immediately afterward, AAV2-EF1a-EGFP, AAV2-EF1a-LoxP-SPRY2-2A-EGFP, and Aflibercept were administered via intravitreal injections to the unilateral eye of all groups, except for the CNV 12d group. On the 12th day after LP treatment, following FFA re-imaging to confirm the therapeutic effects, all experimental animals were sacrificed according to the experimental analysis methods.

### 4.4. FFA

Prior to FFA imaging, mice were anesthetized, and their pupils were dilated. FFA images were taken at appropriate intervals for 5 min following IP injection of 2% (*v*/*v*) fluorescein sodium (Florescite; Akorn, Lake Forest, IL, USA) using a scanning laser ophthalmoscope (Heidelberg Retina Angiography 2; Heidelberg Engineering, Heidelberg, Germany).

FFA images obtained at 5 and 12 days were analyzed using Image J software (ImageJ 1.54g), National Institutes of Health, Bethesda, MD, USA). The maximal border of the hyper-fluorescent area was manually selected for analysis. Area values were converted to mm2, and intensity was calculated in pixels (%) using the scale tool.

### 4.5. Intravitreal Adeno-Associated Virus (AAV) and Aflibercept Administration

Following FFA imaging, the mouse was maintained under anesthesia with dilated pupils. A sharp 30 G needle tip was used to create a sclerotomy approximately 1 mm posterior to the limbus. Subsequently, the drug was injected through the sclerotomy using a NanoFil syringe fitted with a 34 G blunted needle (World Precision Instruments, Inc., Sarasota, FL, USA). During the injection procedure, the retina was directly visualized with a surgical microscope, and a small plastic ring filled with 2% hypromellose was placed on the cornea.

The AAV2-EF1a-LoxP-SPRY2-2A-EGFP viral vector was produced and purified at the Korea Institute of Science and Technology (KIST, Seoul, Republic of Korea) Virus Facility, and injected at a concentration of 1 × 10^9^ GC/uL per eye. AAV2-EGFP was also injected at the same concentration. Aflibercept was injected at a concentration of 40 μg/uL per eye. Both drugs were injected into both eyes of each mouse.

### 4.6. Immunofluorescence

Prior to sacrifice, the mice were deeply anesthetized and perfused through the heart with 0.1 M phosphate buffer (PB) containing 1000 U/mL heparin, followed by perfusion with 4% (*v*/*v*) paraformaldehyde (PFA) in 0.1 M PB (Biosesang, Seongnam, Republic of Korea). After removing the anterior segment to create eyecups from enucleated eyes, they were fixed in 4% PFA at 4 °C for 1 h. The eyecups were dehydrated in 30% (*w*/*v*) sucrose overnight and embedded in a frozen section compound from Leica Biosystems (Richmond, IL, USA). Transverse tissue cryosections of 10 μm were cut and stored at −80 °C until further use.

The slides were dried at 60 °C for 30 min and washed with PB saline (PBS) for 15 min. Permeabilization was achieved by incubating with 0.1% (*v*/*v*) Triton X-100 in PBS for 10 min. Non-specific binding was blocked by incubating the samples with 5% (*v*/*v*) normal donkey serum in PBS for 1 h at room temperature. Subsequently, primary antibodies, diluted in a blocking solution, were applied and incubated overnight at 4 °C. The following day, the slides were washed with PBS and then incubated with secondary antibodies (Alexa Fluor 488 or 568, dilution 1:1000; Invitrogen Corp., Carlsbad, CA, USA) at room temperature for 2 h. Counterstaining was performed using Hoechst^®^ 33342 (Invitrogen, H1399). Finally, the slides were mounted with a mounting medium (Abcam, Cambridge, UK) and imaged using a microscope equipped with a Leica confocal camera (DMI8; Leica Camera, Wetzlar, Germany). All staining procedures were conducted in a dark and humid chamber.

For RPE whole-mount staining, the neuroretina was removed from the RPE/choroid complex. The whole mount was washed with 70% ethanol at room temperature for 30 min, followed by staining using the same procedure described above.

The primary antibodies used included anti-CD31 (BD Biosciences, San Jose, CA, USA), anti-p-p38 (Cell Signaling Technology, Danvers, MA, USA), anti-phosphorylated JNK (p-JNK; Cell Signaling Technology), anti-phosphorylated ERK 1/2 (p-ERK1/2; Cell Signaling Technology), anti-cytokeratin (Alignt Technologies, INC Santa Clara, CA, USA), αSMA (Sigma-Aldrich, St Louis, MO, USA), anti-glial fibrillary acidic protein (GFAP; Abcam, Cambridge, UK), anti-fibronectin (Abcam), anti-β-catenin (Abcam), anti-CD11b (Bio-Rad, Cambridge, UK), and anti-F4/80 (Bio-Rad).

### 4.7. Terminal Deoxynucleotidyl Transferase dUTP Nick End Labeling (TUNEL) Assays

The TUNEL analysis was conducted using the In Situ Cell Death Detection Kit (Sigma-Aldrich, St. Louis, MO, USA), following the manufacturer’s protocol. Cryosections were first washed with PBS and then incubated on ice in a permeabilization solution containing 0.1% (*v*/*v*) Triton X-100 and 0.1% (*w*/*v*) sodium citrate for 3 min. After washing with PBS, the sections were incubated with a mixture of terminal deoxynucleotidyl transferase and fluorescein-labeled deoxyuridine triphosphate at 37 °C for 1 h in a dark and humid chamber.

Following another round of PBS washing, counterstaining was performed, and images were captured using the same microscopy setup as described previously.

### 4.8. Western Blot Analysis

The expression levels of ERK, JNK, p38, p-p38, p-JNK, and p-ERK (1:1000; Cell Signaling Technology), along with β-actin (1:1000; Santa Cruz, Dallas, TX, USA)) in the retina and RPE-choroid complex, were analyzed by Western blotting.

Mice were sacrificed in a CO_2_ chamber under deep anesthesia, and their eyeballs were immersed in PBS to remove the connective tissues, cornea, and optic nerve. After separating the retina and RPE/choroid complex from the eyecup, samples from both eyes were pooled and used for the experiment.

For protein extraction, tissues were homogenized and treated with RIPA II lysis buffer (GenDEPOT, Barker, TX, USA) containing Xpert phosphatase inhibitor cocktail and Xpert protease inhibitor cocktail (GenDEPOT) on ice for 30 min. Following centrifugation at 13,000× *g*, 4 °C, for 15 min, the supernatant containing protein lysate was collected. The protein concentration was determined using the Pierce BCA Protein Assay kit (Thermo Scientific, Middlesex, MA, USA) according to the manufacturer’s instructions. The protein lysate was then dissolved at 1 μg/μL in 5×protein sample buffer (Elpis Biotech, Daejeon, Republic of Korea) and boiled for 10 min at 95 °C. Samples were loaded onto sodium dodecyl sulfate–polyacrylamide gel electrophoresis gels and transferred to polyvinylidene difluoride membranes (Merck, Kenilworth, NJ, USA).

Subsequently, membranes were blocked with 5% (*w*/*v*) skim milk in PBS and incubated on a shaker at room temperature for 1 h. Primary antibodies were incubated overnight at 4 °C on a shaker. The next day, after washing, the membranes were exposed to secondary antibodies conjugated with horseradish peroxidase (GenDEPOT) at room temperature for 2 h. Protein bands were visualized using EzWestLumi plus (ATTO Co., Tokyo, Japan) and imaged with the Azure Biosystems C280 digital system (Azure Biosystems, Dublin, CA, USA). Blot bands were quantified using Image J software (National Institutes of Health).

### 4.9. Voronoi Diagram

To assess the variation in morphology of the RPE cells in the 5 groups of mice, namely the (1) control; (2) CNV12d; (3) CNV 5d + GPF 7d; (4) CNV 5d + SPRY2 7d; (5) CNV 5d + Afilbercept 7d, the Voronoi diagram algorithm was utilized. This was performed using Rhinoceros 5.0 software (Roberts McNeel & Associates, Seattle, WA, USA) on ß-catenin-stained RPE cells [23]. RPE cells with intact ß-catenin staining from five laser-treated mice retinae were averaged for analysis. The mean number and area of RPE cells and coefficients of variance of the areas of RPE cells stained with ß-catenin within 200 µm diameter circles were estimated and calculated using ImageJ software (National Institutes of Health).

### 4.10. Image Analysis and Statistical Analysis

Statistical analyses were performed using SPSS software (version 20.0 for Windows; SPSS Inc., Chicago, IL, USA). The Kruskal–Wallis test with a post hoc analysis was used for multiple comparisons among each time point. A *p*-value of less than 0.05 was considered to indicate statistical significance. All quantified values were expressed as the mean ± standard error of the means.

## Data Availability

The datasets used and/or analyzed during the current study are available from the corresponding author upon reasonable request.

## Supplementary Materials

The following supporting information can be downloaded at https://www.mdpi.com/article/10.3390/ijms26062585/s1.
