# Supplementary material for: The Role of the Mitogen-Activated Protein Kinase Pathway in the Development of Laser-Induced Choroidal Neovascularization
Source: Int J Mol Sci. 2025 Mar 13;26(6):2585. doi: 10.3390/ijms26062585 (PMC11942168; doi:10.3390/ijms26062585)
Supplement: Supplementary file 1 [file ijms-26-02585-s001.zip › ijms-3487293-supplementary.pdf]

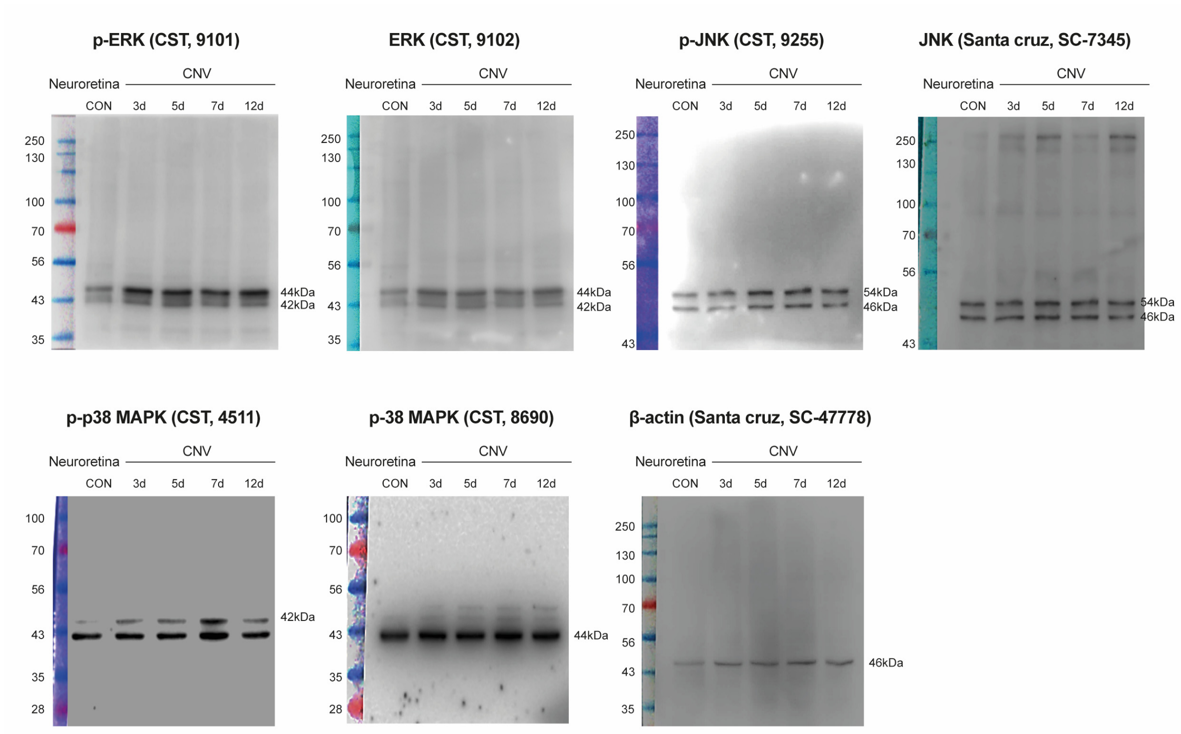

Supplementary Figure S1. Detail of western blots.

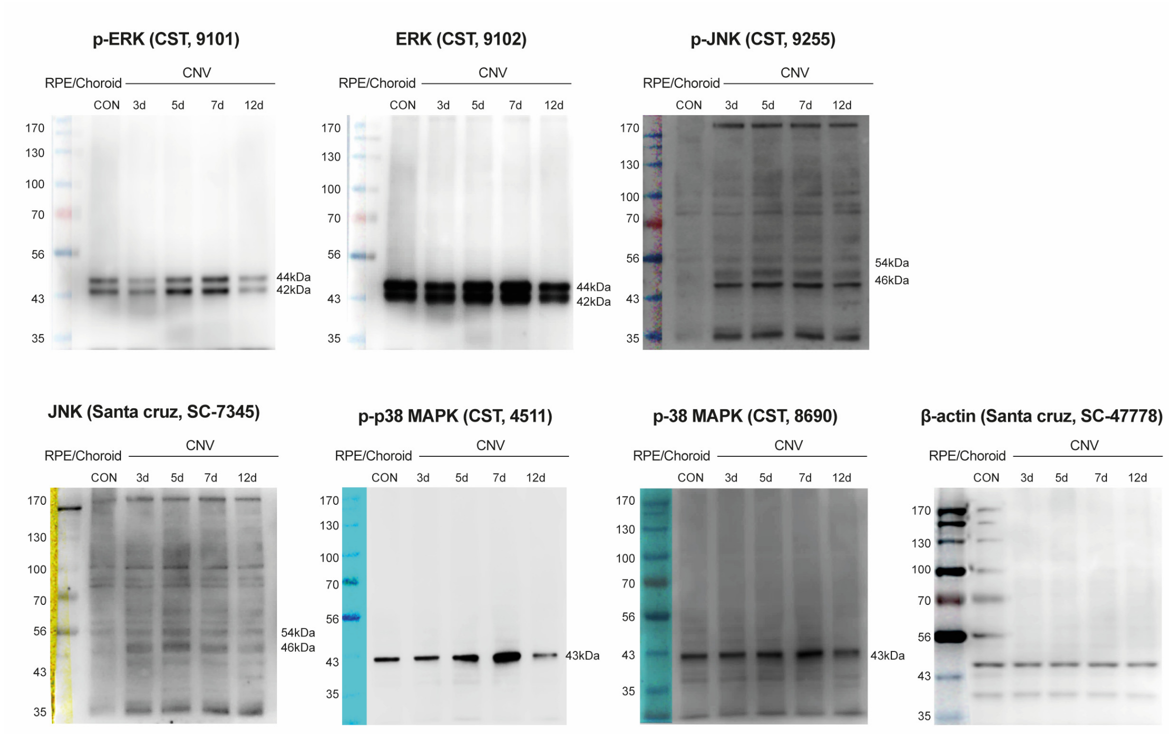

Supplementary Figure S2. Detail of western blots.
